# Effect of Tadalafil Administration on Redox Homeostasis and Polyamine Levels in Healthy Men with High Level of Physical Activity

**DOI:** 10.3390/ijerph18199962

**Published:** 2021-09-22

**Authors:** Guglielmo Duranti, Roberta Ceci, Luigi Di Luigi, Cristina Antinozzi, Ivan Dimauro, Stefania Sabatini, Manuela Cervelli, Paolo Sgrò

**Affiliations:** 1 Laboratory of Biochemistry and Molecular Biology, Department of Movement, Human and Health Sciences, Università degli studi di Roma “Foro Italico”, 00135 Rome, Italy; roberta.ceci@uniroma4.it (R.C.); stefania.sabatini@uniroma4.it (S.S.); 2 Unit of Endocrinology, Department of Movement, Human and Health Sciences, Università degli studi di Roma “Foro Italico”, 00135 Rome, Italy; luigi.diluigi@uniroma4.it (L.D.L.); cristina.antinozzi@uniroma4.it (C.A.); paolo.sgro@uniroma4.it (P.S.); 3 Laboratory of Biology and Genetics, Department of Movement, Human and Health Sciences, Università degli studi di Roma “Foro Italico”, 00135 Rome, Italy; ivan.dimauro@uniroma4.it; 4 Department of Science, “Department of Excellence 2018–2022”, University of Rome “Roma Tre”, 00146 Rome, Italy; manuela.cervelli@uniroma3.it; 5 Neurodevelopment, Neurogenetics and Molecular Neurobiology Unit, IRCCS Fondazione Santa Lucia, Via del Fosso di Fiorano 64, 00143 Rome, Italy

**Keywords:** tadalafil, redox homeostasis, polyamines, antioxidant enzyme activities

## Abstract

*Background:* The phosphodiesterase type 5 inhibitor (PDE5I) tadalafil, in addition to its therapeutic role, has shown antioxidant effects in different in vivo models. Supplementation with antioxidants has received interest as a suitable tool for preventing or reducing exercise-related oxidative stress, possibly leading to the improvement of sport performance in athletes. However, the use/abuse of these substances must be evaluated not only within the context of amateur sport, but especially in competitions where elite athletes are more exposed to stressful physical practice. To date, very few human studies have addressed the influence of the administration of PDE5Is on redox balance in subjects with a fitness level comparable to elite athletes; therefore, the aim of this study was to investigate for the first time whether acute ingestion of tadalafil could affect plasma markers related to cellular damage, redox homeostasis, and blood polyamines levels in healthy subjects with an elevated cardiorespiratory fitness level. *Methods:* Healthy male volunteers (n = 12), with a VO_2max_ range of 40.1–56.0 mL/(kg × min), were administered with a single dose of tadalafil (20 mg). Plasma molecules related to muscle damage and redox-homeostasis, such as creatine kinase (CK), lactate dehydrogenase (LDH), total antioxidant capacity (TAC), reduced/oxidized glutathione ratio (GSH/GSSG), free thiols (FTH), antioxidant enzyme activities (superoxide dismutase (SOD), catalase (CAT) and glutathione peroxidase (GPx)), as well as thiobarbituric acid reactive substances (TBARs), protein carbonyls (PrCAR), and polyamine levels (spermine (Spm) and spermidine (Spd)) were evaluated immediately before and 2, 6 and 24 hours after the acute tadalafil administration. *Results:* A single tadalafil administration induced an increase in CK and LDH plasma levels 24 after consumption. No effects were observed on redox homeostasis or antioxidant enzyme activities, and neither were they observed on the oxidation target molecules or polyamines levels. *Conclusion:* Our results show that in subjects with an elevated fitness level, a single administration of tadalafil induced a significant increase in muscle damage target without affecting plasma antioxidant status.

## 1. Introduction

Phosphodiesterase type 5 inhibitors (PDE5Is) (e.g., avanafil, sildenafil, vardenafil, tadalafil), represent a class of drugs routinely consumed worldwide, because of their great therapeutic role for the treatment of erectile dysfunction (ED) (avanafil, sildenafil, vardenafil, tadalafil), and pulmonary arterial hypertension (sildenafil and tadalafil), and for the management, in men, of moderate to severe lower urinary tract symptoms (LUTS) secondary to benign prostatic obstruction with or without ED (tadalafil) acting mainly via the nitric oxide (NO) and cyclic guanosine monophosphate (NO–cGMP) pathways [1,2,3,4,5,6,7,8].

Recently, in addition to their role in the treatment of pathologies, in vitro and in vivo studies reported that PDE5Is (e.g., sildenafil, vardenafil, tadalafil) also possess antioxidant effects in different tissues/organs [9,10,11,12,13,14]. For instance, it has been shown that sildenafil citrate has protective effects against oxidative stress by inhibiting free radical formation and supporting antioxidant redox systems [13,15]. Interestingly, in a model of a fetal rat brain with an ischemia/reperfusion injury, tadalafil was found to be more effective than sildenafil in reducing the endogenous superoxide formation by increasing the antioxidant enzyme activity of glutathione peroxidase (GPx) [16].

To date, the capacity of tadalafil to improve mitochondrial integrity, oxidative metabolism, and antioxidant activity (i.e., superoxide dismutases (SODs) and GPx) was also observed in physio-pathological conditions using either animal models [11,17,18,19,20,21,22] or cell cultures [23,24] challenged with a pro-oxidant stimulus. The general picture that emerges from these studies suggests that PDE5Is could represent new antioxidant molecules.

Supplementation with antioxidants and/or drugs with antioxidant potential has received interest as a suitable tool for preventing or reducing exercise-related oxidative stress, possibly leading to the improvement of sport performance in athletes. Indeed, there are an increasing number of athletes (≈88%) using one or more nutritional supplements [25]. However, special attention must be given to reduce the risk which comes from the improper use of these substances, limiting their use to only specific cases/conditions.

It is surprising to note that although there is much evidence to support the antioxidant effects of PDE5Is in animal models, to our knowledge, there is little information about the influence of tadalafil administration on redox balance in humans.

In 2015, our group highlighted the potential negative effects of tadalafil in healthy males [26]. In particular, we found that a prolonged exposure (repeated intake) to tadalafil increased plasma creatine kinase (CK) and lactate dehydrogenase (LDH) plasma levels, and decreased antioxidant capacity at resting conditions, thereby making subjects more susceptible to the oxidative stress induced by an exhaustive bout of exercise [26].

The purpose of this study was to investigate for the first time the effect of a single dose of tadalafil on the oxidant/antioxidant status and polyamines (PAs) levels in subjects with a cardiovascular fitness comparable to that of elite athletes and/or well-trained subjects, a group of individuals continuously exposed to exercise-induced oxidative stress.

To this end, blood sample collections were performed at different time points from tadalafil administration in a double-blind protocol to evaluate muscle damage target molecules, redox homeostasis, oxidation target molecules, antioxidant enzyme activities and PAs levels.

## 2. Materials and Methods

### 2.1. Subjects

The study was carried out on blood samples collected from twelve healthy men with high levels of physical activity (n = 12), with a VO_2max_ range of 40.1–56.0 mL/(kg × min) (Table 1) (Di Luigi et al., 2008 [27]). All the subjects were involved in university athletic activity and in team sports (soccer, basketball, volleyball). The volunteers were non-smoker subjects who usually took no medication, anabolic agents, dietary supplementations, amino acid supplementation (e.g., arginine), vitamins and/or antioxidants. Ethical Committee’s approval (Policlinico Umberto I–p.1039/08) and written informed consent were obtained. Moreover, they completed a detailed eating habits diary in which all food and drinks consumed were recorded.

### 2.2. Experimental Protocol

**Figure 1:**
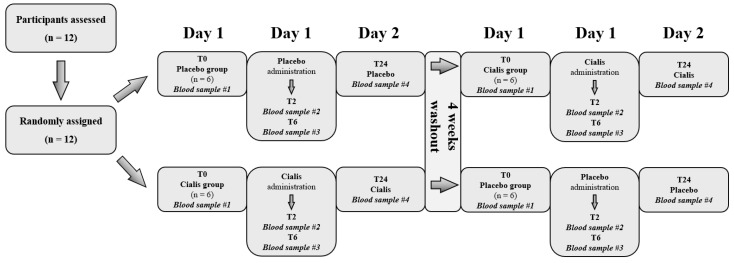
Flow diagram of the study. Twelve subjects were selected and then randomly assigned to the Cialis group (n = 6) or to the placebo group (n = 6) (Day 1). Then, subjects were given either Cialis (one tablet) or the placebo. Blood collections were performed at baseline (T0), and 2, 6 and 24 hours (Day 2) after administration (T2-T6-T24). After 4 weeks of washout, in the second experimental session, the volunteers were switched, and received either the placebo or Cialis, respectively. Each subject was his own control.

After 10 h overnight fasting and no consumption of caffeine for the prior 24 h, all the volunteers randomly received (at 8:00 a.m.) one tablet of tadalafil per os (20 mg, Cialis^®^, Ely-Lilly, Indianapolis, IN, USA) in a double-blind experimental protocol (Figure 1). No exercise, sexual intercourse, or major stress events were allowed, starting from 48 h before, and then continuing throughout, the protocol.

### 2.3. Blood Samples Collection

Blood sample collections (10 mL for each draw) were performed immediately before (T0), and at 2, 6, and 24 hours after tadalafil administration (T2, T6 and T24, respectively). Plasma was immediately separated (3000 rpm, 10 min, +4 °C) and stored at −80 °C until biochemical assays.

### 2.4. Biochemical Analysis

On blood samples collected, we evaluated: (1) muscle damage target molecules (creatine kinase (CK), lactate dehydrogenase (LDH)); (2) redox homeostasis (total antioxidant capacity (TAC), total glutathione (tGSH), reduced/oxidized glutathione ratio (GSH/GSSG), plasma free thiols); (3) oxidation target molecules (malondialdehyde (TBARs), protein carbonyls (oxidized proteins)); (4) antioxidant enzyme activities (superoxide dismutase (SOD), catalase (CAT) and glutathione peroxidase (GPx)); (5) polyamine levels (spermidine (Spd), spermine (Spm)).

#### 2.4.1. Materials

All chemical reagents, unless specified otherwise, were purchased from Sigma-Aldrich Chemical (St. Louis, MO, USA).

#### 2.4.2. Creatine Kinase and Lactate Dehydrogenase

Plasma creatine kinase (CK) and lactate dehydrogenase (LDH) levels were measured as previously described [28] using commercial assay kits (Greiner Diagnostic GmbH, Bahlingen, Germany) following the manufacturer’s instructions. Results were expressed as U Enz/L.

#### 2.4.3. Glutathione Homeostasis

Plasma reduced (GSH) and oxidized (GSSG) glutathione content was quantified by a 5,5-dithio-bis-(2-nitrobenzoic acid) (DTNB)-glutathione reductase recycling assay as previously described [29].

#### 2.4.4. Total Plasma Free Thiol Determination

The free thiol (FTH) concentration of plasma samples was quantified by the Ellman assay according to Colombo et al. [30]. The molar concentration of thiols was calculated from the molar absorbance of the 2-nitro-5-thiobenzoate (TNB) anion and expressed as µmol-SH/g plasmatic proteins.

#### 2.4.5. Trolox Equivalents Antioxidant Capacity (TAC)

Plasma TAC was determined spectrophotometrically, according to Ceci et al. [31]. This method is based on the reactivity of plasmatic antioxidant compounds relative to a 1 mM Trolox^®^ (vitamin E analog) standard.

In vitro tadalafil (10–150 µM) and ascorbate (12.5–100 µM) TAC were also assayed in order to evaluate their ability to prevent 2,2’-azino-bis-(3-ethylbenzothiazoline-6-sulfonic acid) radical formation (ABTS°+).

The variation of absorbance detected was compared to those obtained using Trolox^®^ standards.

#### 2.4.6. Thiobarbituric Acid Reactive Substances (TBARs)

TBARs level were assayed by spectrophotometric analysis [32]. The methodology measures malondialdehyde and other aldehydes produced by lipid peroxidation induced by hydroxyl free radicals. The levels of TBARs were expressed in terms of µmol/L.

#### 2.4.7. Protein Carbonyls (PrCar)

Protein carbonyl levels were determined by measuring the reactivity of carbonyl derivatives with 2,4-dinitrophenylhydrazine (DNPH) as previously described [33]. Protein carbonyl content was expressed in terms of nmol/mg protein.

#### 2.4.8. Enzymatic Activities

Intracellular superoxide dismutase (SOD), catalase (CAT) and glutathione peroxidase (GPx) activities were measured as previously described [24] using commercial assay kits (Cayman Chemical Company, Ann Arbor, MI, USA) following the manufacturer’s instructions. Results were expressed as units/mg of protein tested.

#### 2.4.9. Polyamines Levels

Polyamines levels (spermidine (Spd), spermine (Spm), and relative ratio (Spm/Spd)) were determined as previously described by HPLC with fluorimetric analysis [34].

### 2.5. Statistical Analyses

The values were expressed as the means ± standard deviations (SD). Statistical analyses were performed using SPSS software (Version 21.0, IBM SPSS Statistics, Armonk, NY, USA). An a priori analysis (GPower 3.1, Heinrich-Heine-Universität Düsseldorf, Dusseldorf, Germany) was used to determine a sample size that yielded power values of 0.8 or greater. The Kolmogorov–Smirnov test was used to evaluate the variable distribution. A one-way ANOVA for repeated measures and Bonferroni post hoc analyses were used to determine significant variations over time for each parameter evaluated. A *p*-value <0.05 was considered statistically significant in all analysis.

No statistically significant changes were found for all parameters analyzed in the placebo group. All the data shown refer to the values found in the Cialis group.

## 3. Results

### 3.1. Baseline Characteristics of Subjects

Healthy and physically active subjects were recruited with a fitness level between the “excellence” and “superior” range (47.7 ± 4.9 mL/(kg × min) (40.1–56.0) VO_2max_, Table 1) [35,36].

### 3.2. Creatine Kinase and Lactate Dehydrogenase Analysis

A single tadalafil administration was able to increase plasma markers of muscle damage only after 24 h from ingestion. CK (39.70 ± 6.80 vs. 28.31 ± 7.08, *p* < 0.01) and LDH (49.53 ± 8.43 vs. 40.90 ± 6.90, *p* < 0.05) levels resulted in increased respect T0 (Table 2).

### 3.3. Glutathione Homeostasis

No statistically significant variations in tGSH values and in the GSH/GSSG ratio were found after a single tadalafil administration (Table 3).

On the other hand, an increase in oxidized glutathione was found after 6 hours (7.13 ± 4.16 vs. 5.64 ± 3.08, *p* < 0.05) and maintained after 24 hours (7.07 ± 3.67 vs. 5.64 ± 3.08, *p* < 0.05).

### 3.4. Total Plasma Free Thiol Determination and Trolox Equivalents Antioxidant Capacity

A time-dependent analysis showed no statistically significant variation in FTH and TAC values after a single tadalafil administration (Table 3).

### 3.5. Oxidative Damage Analysis

Single tadalafil administration induced no statistically significant variation in plasma TBARs and PrCAR levels (Table 4).

### 3.6. Enzymatic Activities

A time-dependent analysis showed no statistically significant variation in plasma CAT, SOD, GPx levels and SOD/CAT and SOD/GPx ratios after a single tadalafil administration (Table 5).

### 3.7. Polyamines Levels

Single tadalafil administration induced no statistically significant variation in blood Spm and Spd levels and Spm/Spd ratio (Table 6).

## 4. Discussion

The aim of this study was to assess the effects of an acute tadalafil administration on markers of muscle damage and oxidative balance in healthy men with an elevated cardiorespiratory fitness level. Indeed, all subjects have a VO_2max_ between 40.1 and 56.0 mL/(kg × min), corresponding for the chronological age to an excellent/superior level of cardiorespiratory fitness (Table 1).

Although the issue is still controversial, active individuals (e.g., athletes and well-trained subjects) often consume antioxidants to provide protection against oxidative stress and reduce muscular damage and fatigue symptoms [37,38,39,40].

Generally speaking, there is an increasing demand for molecules or drugs capable of managing physical and oxidative stress. This fact entails a continuous attention to the phenomenon of the unauthorized abuse of these compounds.

In this context, a great interest has shifted towards PDE5Is. Many healthy athletes who participate in sports which require endurance, and/or who compete in hypoxic conditions, misuse PDE5Is to improve performance, as these substances are not prohibited by the World Anti-Doping Agency (WADA) [41,42].

These compounds have recently showed antioxidant effects in different organs of animal models. This effect is achieved through the reduction of endogenous superoxide formation and the increase in the antioxidant enzymes’ activity [9,10,11,13,15,16]. Moreover, an improved mitochondrial integrity [11,21], a decreased tissue malondialdehyde and increased SOD and GPx activity levels were found following tadalafil administration [17,18,19,20,21,22]. In a mouse model of contrast-induced nephropathy, sildenafil and tadalafil pretreatment were able to reduce nephropathy risk and reversed oxidative stress by modulating the oxidant/antioxidant balance [8].

To focus on the human model, we have demonstrated that in healthy males, a prolonged exposure to tadalafil decreases antioxidant capacity by reducing the GSH/GSSG ratio and increasing TBARs and PrCAR levels [26]. Therefore, these results suggest that the subjects have a greater susceptibility to oxidative stress. This aspect is particularly important considering that they were subjects with a high level of fitness.

To better understand the effects of tadalafil on well-trained subjects, we verified if a single dose of tadalafil increased plasma markers of muscle damage. We found that 24 hours after tadalafil ingestion, creatine kinase and lactate dehydrogenase plasma levels resulted in increased respect T0 (Table 2). These data support the hypothesis that the evidenced side effects (i.e., back pain) of the drug administration [43] could be due to its action on muscle tissue.

Subsequently, we wondered if the increase in circulating CK and LDH was paralleled by an altered redox homeostasis.

Glutathione is the primary antioxidant responsible for maintaining a reduced intracellular microenvironment. When reactive oxygen species (ROS) production is accelerated to the point that it overwhelms ROS scavenging capacity, the GSH/GSSG ratio, a well-known marker of oxidative stress, decreases, significantly reducing the antioxidant capacity [24].

In our healthy subjects, we found that a single pill substantially unaffected plasma redox homeostasis, as evidenced by glutathione levels analysis (Table 3).

An increase of 31.5 and 27.5% of GSSG were found after 6 and 24 hours with respect T0. However, this increase is not reflected in a significant decrease in the GSH/GSSG ratio, thus indicating that the buffering capacity of the plasma is able to maintain the right redox state.

In plasma, protein thiols act as a redox buffer; their concentration is much greater than those of low-molecular weight thiols. The oxidation of protein thiols to mixed disulfides is an early response to oxidative stress [44].

In our subjects, plasma free thiols remained unchanged by acute tadalafil administration, indicating that no effect on thiol/disulfide status in plasma occurred (Table 3).

Regarding the oxidative damage, the time and dose of administration of tadalafil seems to cause different effects in healthy humans. We demonstrated in a previous paper that a prolonged (two tablets with 36 hours of interval) tadalafil ingestion caused an increase in malondialdehyde (TBARs) and protein carbonylation. However, here we demonstrated that an acute dose (single administration) did not induce an increase in oxidation target molecules (Table 4).

It is known that PDE5Is modulate antioxidant enzyme activities systems. The antioxidant enzymes’ superoxide dismutase (SOD) and glutathione peroxidase (GPx) activity levels increased after administration and following a stressful stimulus [16,19,20]. Sildenafil citrate resulted in a significant increase in the erythrocyte superoxide dismutase and catalase activities in humans [15]. Moreover, recently, we have demonstrated that tadalafil may be beneficial to skeletal muscle cells by enhancing the enzymatic antioxidant system capacity in C2C12 skeletal muscle cells [24].

Nevertheless, analysis of plasma antioxidant enzyme activities revealed that, in the subjects analyzed, a single dose of tadalafil was not able to induce superoxide dismutase (SOD), catalase (CAT) and glutathione peroxidase (GPx) activities. Hence, during acute treatment, SOD, CAT and GPx plasma levels do not reflect the presumed intracellular increase, so we can speculate that tadalafil has a putative beneficial effect for improving cellular enzymatic antioxidant system capacity without evidence at systemic level (Table 5).

Polyamines (PAs) are naturally occurring aliphatic compounds that are intrinsic constituents of all eukaryotic cells. Among these, spermine (Spm) and spermidine (Spd) play essential roles in many cellular functions [45,46,47,48] and possess different functions in the protection from reactive oxygen species [49]. Several in vitro studies have demonstrated that PAs protect cells from hydrogen peroxide- and superoxide-induced oxidative stress. Interestingly their effect seems to be distinct from that played by the most important primary antioxidant glutathione [49].

Polyamines are amines derived from arginine, which is the precursor of nitric oxide (NO) [47]. An alteration in PAs levels may affect the NO bioavailability due to the close relationship between PAs and NO metabolism [49].

In subjects who have taken a pill of tadalafil, the persistence of NO, due to phosphodiesterase type 5 (PDE5) inhibition, could determine a greater ability for this molecule to react with ROS to form toxic peroxynitrite [50]. These molecules represent the most reactive free radical species [51], able to induce a redox unbalance at rest.

It was demonstrated that PAs levels were increased in childhood obesity and correlated to markers of oxidative/nitrosative stress [52].

Interestingly, in our subjects, blood Spd and Spm levels remained unchanged by acute tadalafil administration, indicating that the increase in NO bioavailability substantially unaffected PAs metabolism (Table 6).

Finally, plasma’s total antioxidant capacity, which expressed the reactivity of plasmatic antioxidant compounds in counteracting radicals, also remained unchanged. In vitro analysis of the scavenger capacity of tadalafil showed modest activity (Table 7) and, therefore, although the molecule may act for longer than other PDE5 inhibitors [6], its contribution to the plasma total antioxidant capacity may be not significant.

There are few studies evaluating the effects of tadalafil on healthy athletes [27,53,54,55]. Many studies have been conducted on subjects with pathologies or who are in conditions of extreme physiological stress [56,57,58,59,60,61,62,63]. Recently, using a similar group of subjects, we have demonstrated that a single dose of tadalafil did not substantially influence performance indicators, during a maximal standardized exercise test in a healthy athlete [27]. Moreover, the consumption did not modify the time to peak power and increased blood lactate concentrations during recovery from exercise [55]. This effect could be related to a possible effect of PDE5Is in stimulating anaerobic glycolysis [23].

The particular category of subjects used in this study could explain the results obtained here. In fact, in the athletes/well-trained subjects with the highest cardiovascular fitness levels, there is a continuous physiological adaptation responding to oxidative stress, which is especially realized in the increase in the antioxidant systems and in the control of the redox state [31,64,65,66].

## 5. Conclusions

In the context of an increased demand for molecules or drugs that can help people to sustain physical and oxidative stress, it is especially important to know whether the use/abuse of substances may be safe or not. Although further studies are needed to clarify the molecular mechanisms behind these results, here, we showed for the first time that a single dose of tadalafil increases the plasma levels of muscle damage markers without affecting antioxidant status related molecules in healthy men with high levels of physical activity.

It must be considered that our results cannot be generalized to the entire sporting population. Indeed, we cannot exclude that in subjects with low levels of physical activity, and, therefore, with a less adapted redox system, tadalafil could have a positive effect.

Nevertheless, as with other drugs with antioxidant properties, the initial antioxidant values should be considered, as well as the possible relationship among redox biomarkers, measured before and after exercise-induced redox challenge in subjects who consumed these substances. Moreover, these data could be useful to identify the right dose, thus avoiding the possible negative effects (e.g., muscle damage) which can derive from a high dosage of these molecules.

## Figures and Tables

**Table 1:** Subjects’ characteristics: body mass index (BMI); maximal oxygen uptake (VO_2max_).

Subjects’ Characteristics (n = 12)	
Age (years)	24.4 ± 3.4; (19–31)
Height (cm)	172.2 ± 6.7; (157.0–184.0)
Weight (kg)	73.8 ± 5.2; (65.0–84.0)
BMI (kg m^−2^)	24.9 ± 1.8; (22.0–28.4)
VO_2max_ (mL min^−1^ kg^−1^)	47.7 ± 4.9; (40.1–56.0)

BMI: Body Mass Index; VO_2max_: maximal oxygen consumption. Data are expressed as mean ± SD and respective range of values (min–max).

**Table 2:** Plasma creatine kinase and lactate dehydrogenase levels.

Variables	T0	T2	T6	T24
CK ^a^	28.31 ± 7.08	29.76 ± 6.67	33.57 ± 6.43	39.70 ± 6.80 ^#^
LDH ^a^	40.90 ± 6.90	42.22 ± 7.02	45.41 ± 6.85	49.53 ± 8.43 *

Creatine kinase (CK) and lactate dehydrogenase (LDH) levels were measured in plasma before (T0) and 2, 6 and 24 hours (T2–T24) after tadalafil administration. Data presented are the mean ± SD, n = 12, performed in triplicate. ^#^ *p* < 0.01 vs. T0; * *p* < 0.05 vs. T0. ^a^ U Enz/L.

**Table 3:** Plasma glutathione homeostasis, free thiols levels and total antioxidant capacity.

Variables	T0	T2	T6	T24
Total GSH ^a^	100.48 ± 32.50	99.23 ± 33.24	102.37 ± 30.32	104.46 ± 31.38
GSSG ^a^	5.64 ± 3.08	6.43 ± 3.39	7.13 ± 4.16 *	7.07 ± 3.67 **
GSH/GSSG	21.52 ± 15.12	16.65 ± 5.86	17.54 ± 12.34	20.02 ± 20.41
FTH ^b^	3.28 ± 0.39	3.20 ± 0.31	3.16 ± 0.34	3.26 ± 0.43
TAC ^c^	0.77 ± 0.11	0.74 ± 0.11	0.75 ± 0.11	0.75 ± 0.13

Plasma total glutathione, oxidized glutathione (GSSG) and reduced (GSH) to oxidized glutathione ratio (GSH/GSSG), free thiols (FTH) levels and total antioxidant capacity (TAC) were measured in plasma before (T0) and 2, 6 and 24 hours (T2–T24) after tadalafil administration. Data presented are the mean ± SD, n = 12, performed in triplicate. ^a^ 10^−5^ M; ^b^ µmol-SH/g; ^c^ Trolox^®^ equivalents mM; * +31.3% *p* < 0.05 vs. T0; ** +27.5% *p* < 0.05 vs. T0.

**Table 4:** Plasma thiobarbituric acid reactive substances and protein carbonyl levels.

Variables	T0	T2	T6	T24
TBARs ^a^	0.72 ± 0.08	0.75 ± 0.04	0.67 ± 0.05	0.68 ± 0.07
PrCAR ^b^	1.63 ± 0.08	1.65 ± 0.10	1.67 ± 0.08	1.56 ± 0.08

Plasma thiobarbituric acid reactive substances (TBARs) and protein carbonyl (PrCAR) levels were measured in plasma before (T0) and 2, 6 and 24 hours (T2–T24) after tadalafil administration. Data presented are the mean ± SD, n = 12, performed in triplicate. ^a^ µmol/L; ^b^ nmol/mg.

**Table 5:** Plasma catalase, superoxide dismutase and glutathione peroxidase activities analysis.

Variables	T0	T2	T6	T24
CAT ^a^	19.13 ± 1.81	20.12 ± 1.35	20.87 ± 1.62	20.02 ± 2.09
SOD ^a^	4.72 ± 0.70	5.05 ± 0.91	5.09 ± 1.38	5.04 ± 1.00
GPx ^a^	9.62 ± 1.38	9.89 ± 1.81	9.93 ± 1.67	10.19 ± 2.16
SOD/CAT	0.25 ± 0.04	0.25 ± 0.05	0.24 ± 0.07	0.25 ± 0.05
SOD/GPx	0.50 ± 0.11	0.53 ± 0.13	0.52 ± 0.14	0.51 ± 0.13

Plasma catalase (CAT), superoxide dismutase (SOD), glutathione peroxidase (GPx) activities and SOD/CAT and SOD/GPx ratio analysis were measured in plasma before (T0) and 2, 6 and 24 hours (T2–T24) after tadalafil administration. Data presented are the mean ± SD, n = 12, performed in triplicate, ^a^ U/mL.

**Table 6:** Blood spermidine and spermidine levels.

Variables	T0	T2	T6	T24
Spd ^a^	22.36 ± 12.47	18.06 ± 6.39	23.13 ± 11.16	20.38 ± 8.16
Spm ^a^	17.04 ± 7.36	15.24 ± 5.09	19.55 ± 7.48	15.99 ± 4.78
Spd/Spm	1.29 ± 0.39	1.26 ± 0.43	1.20 ± 0.40	1.31 ± 0.49

Plasma spermidine (Spd), spermine (Spm) and relative ratios (Spd/Spm) analysis were measured in blood before (T0) and 2, 6 and 24 hours (T2–T24) after tadalafil administration. Data presented are the mean ± SD, n = 12, performed in triplicate. ^a^ nmol/mL.

**Table 7:** Tadalafil total antioxidant capacity.

Variables and Groups		1 µM	2 µM	10 µM	100 µM
TAC (Trolox^®^ equivalents mM)	TAD	0.079 ± 0.008	0.082 ± 0.012	0.109 ± 0.011	0.385 ± 0.106
	AA	1.251 ± 0.079	1.796 ± 0.091	2.165 ± 0.012	2.816 ± 0.016

Tadalafil (1–100 µM) in vitro modified Trolox^®^ equivalent antioxidant capacity assay. Data presented as Trolox^®^ millimolar equivalents are the mean ± SD of three experiments. TAD: Tadalafil; AA: Sodium ascorbate (1–100 µM) was used as reference.

## Data Availability

The data presented in this study are available on request from the corresponding author. The data are not publicly available due to privacy or ethical restrictions.

## References

[1] Corbin J.D., Francis S.H. (2002). Pharmacology of phosphodiesterase-5 inhibitors. Int. J. Clin. Pract..

[2] Wright P.J. (2006). Comparison of phosphodiesterase type 5 (PDE5) inhibitors. Int. J. Clin. Pract..

[3] Salloum F.N., Chau V.Q., Hoke N.N., Abbate A., Varma A., Ockaili R.A., Toldo S., Kukreja R.C. (2009). Phosphodiesterase-5 inhibitor, tadalafil, protects against myocardial ischemia/reperfusion through protein-kinase g-dependent generation of hydrogen sulfide. Circulation.

[4] Francis S.H., Busch J.L., Corbin J.D., Sibley D. (2010). cGMP-dependent protein kinases and cGMP phosphodiesterases in nitric oxide and cGMP action. Pharmacol. Rev..

[5] Vachiery J.L., Huez S., Gillies H., Layton G., Hayashi N., Gao X., Naeije R. (2011). Safety, tolerability and pharmacokinetics of an intravenous bolus of sildenafil in patients with pulmonary arterial hypertension. Br. J. Clin. Pharmacol..

[6] Di Luigi L., Sansone M., Sansone A., Ceci R., Duranti G., Borrione P., Crescioli C., Sgrò P., Sabatini S. (2017). Phosphodiesterase Type 5 Inhibitors, Sport and Doping. Curr. Sports Med. Rep..

[7] Georgiadis G., Zisis I.E., Docea A.O., Tsarouhas K., Fragkiadoulaki I., Mavridis C., Karavitakis M., Stratakis S., Stylianou K., Tsitsimpikou C. (2020). Current Concepts on the Reno-Protective Effects of Phosphodiesterase 5 Inhibitors in Acute Kidney Injury: Systematic Search and Review. J. Clin. Med..

[8] Iordache A.M., Docea A.O., Buga A.M., Zlatian O., Ciurea M.E., Rogoveanu O.C., Burada F., Sosoi S., Mitrut R., Mamoulakis C. (2020). Sildenafil and tadalafil reduce the risk of contrast-induced nephropathy by modulating the oxidant/antioxidant balance in a murine model. Food Chem. Toxicol..

[9] Morano S., Mandosi E., Fallarino M., Gatti A., Tiberti C., Sensi M., Gandini L., Buchetti B., Lenti L., Jannini E.A. (2007). Antioxidant treatment associated with sildenafil reduces monocyte activation and markers of endothelial damage in patients with diabetic erectile dysfunction: A double-blind, placebo-controlled study. Eur. Urol..

[10] Fan Y.F., Zhang R., Jiang X., Wen L., Wu D.C., Liu D., Yuan P., Wang Y.L., Jing Z.C. (2013). The phosphodiesterase-5 inhibitor vardenafil reduces oxidative stress while reversing pulmonary arterial hypertension. Cardiovasc. Res..

[11] Koka S., Das A., Salloum F.N., Kukreja R.C. (2013). Phosphodiesterase-5 inhibitor tadalafil attenuates oxidative stress and protects against myocardial ischemia/reperfusion injury in type 2 diabetic mice. Free Radic. Biol. Med..

[12] Antinozzi C., Sgrò P., Marampon F., Caporossi D., Del Galdo F., Dimauro I., Di Luigi L. (2021). Sildenafil Counteracts the In Vitro Activation of CXCL-9, CXCL-10 and CXCL-11/CXCR3 Axis Induced by Reactive Oxygen Species in Scleroderma Fibroblasts. Biology.

[13] Di Luigi L., Duranti G., Antonioni A., Sgrò P., Ceci R., Crescioli C., Sabatini S., Lenzi A., Caporossi D., Del Galdo F. (2020). The Phosphodiesterase Type 5 Inhibitor Sildenafil Improves DNA Stability and Redox Homeostasis in Systemic Sclerosis Fibroblasts Exposed to Reactive Oxygen Species. Antioxidants.

[14] Di Luigi L., Sgrò P., Duranti G., Sabatini S., Caporossi D., Del Galdo F., Dimauro I., Antinozzi C. (2020). Sildenafil Reduces Expression and Release of IL-6 and IL-8 Induced by Reactive Oxygen Species in Systemic Sclerosis Fibroblasts. Int. J. Mol. Sci..

[15] Perk H., Armagan A., Naziroğlu M., Soyupek S., Hoscan M.B., Sütcü R., Ozorak A., Delibas N. (2008). Sildenafil citrate as a phosphodiesterase inhibitor has an antioxidant effect in the blood of men. J. Clin. Pharm. Ther..

[16] Ozdegirmenci O., Kucukozkan T., Akdag E., Topal T., Haberal A., Kayir H., Oter S., Akyol M., Uzbay T. (2011). Effects of sildenafil and tadalafil on ischemia/reperfusion injury in fetal rat brain. J. Matern. Fetal Neonatal Med..

[17] Speranza L., Franceschelli S., Pesce M., Vinciguerra I., De Lutiis M.A., Grilli A., Felaco M., Patruno A. (2008). Phosphodiesterase type-5 inhibitor and oxidative stress. Int. J. Immunopathol. Pharmacol..

[18] Arikan D.C., Bakan V., Kurutas E.B., Sayar H., Coskun A. (2010). Protective effect of tadalafil on ischemia/reperfusion injury of rat ovary. J. Pediatr. Surg..

[19] Chen Y., Li X.X., Lin H.C., Qiu X.F., Gao J., Dai Y.T., Wang R. (2012). The effects of long-term administration of tadalafil on STZ-induced diabetic rats with erectile dysfunction via a local antioxidative mechanism. Asian J. Androl..

[20] Mostafa T., Rashed L., Kotb K., Taymour M. (2012). Effect of testosterone and frequent low-dose sildenafil/tadalafil on cavernous tissue oxidative stress of aged diabetic rats. Andrologia.

[21] Koka S., Aluri H.S., Xi L., Lesnefsky E.J., Kukreja R.C. (2014). Chronic inhibition of phosphodiesterase 5 with tadalafil attenuates mitochondrial dysfunction in type 2 diabetic hearts: Potential role of NO/SIRT1/PGC-1α signaling. Am. J. Physiol. Heart Circ. Physiol..

[22] Adeneye A.A., Benebo A.S. (2016). Chemopreventive Effect of Tadalafil in Cisplatin-Induced Nephrotoxicity in Rats. Niger. J. Physiol. Sci..

[23] Sabatini S., Sgrò P., Duranti G., Ceci R., Di Luigi L. (2011). Tadalafil alters energy metabolism in C2C12 skeletal muscle cells. Acta Biochim. Pol..

[24] Duranti G., Ceci R., Sgrò P., Sabatini S., Di Luigi L. (2017). Influence of the PDE5 inhibitor tadalafil on redox status and antioxidant defense system in C2C12 skeletal muscle cells. Cell stress and chaperones. Cell Stress Chaperones.

[25] Mazzeo F., Tafuri D., Vasilescu M., Ionescu Anca M. (2014). Pharmacologically active substances and dietary supplements used by athletes - the European and Italian regulation. Med. Sport.

[26] Ceci R., Duranti G., Sgrò P., Sansone M., Guidetti L., Baldari C., Sabatini S., Di Luigi L. (2015). Effects of tadalafil administration on plasma markers of exercise-induced muscle damage, IL6 and antioxidant status capacity. Eur. J. Appl. Physiol..

[27] Di Luigi L., Baldari C., Pigozzi F., Emerenziani G.P., Gallotta M.C., Iellamo F., Ciminelli E., Sgrò P., Romanelli F., Lenzi A. (2008). The long-acting phosphodiesterase inhibitor tadalafil does not influence athletes’ VO2max, aerobic, and anaerobic thresholds in normoxia. Int. J. Sports Med..

[28] Duranti G., La Rosa P., Dimauro I., Wannenes F., Bonini S., Sabatini S., Parisi P., Caporossi D. (2011). Effects of salmeterol on skeletal muscle cells: Metabolic and proapoptotic features. Med. Sci. Sports Exerc..

[29] Colamartino M., Duranti G., Ceci R., Sabatini S., Testa A., Cozzi R. (2017). A multi-biomarker analysis of the antioxidant efficacy of Parkinson’s disease therapy. Toxicol. In Vitro.

[30] Colombo G., Reggiani F., Podestà M.A., Garavaglia M.L., Portinaro N.M., Milzani A., Badalamenti S., Dalle-Donne I. (2015). Plasma protein thiolation index (PTI) as a biomarker of thiol-specific oxidative stress in haemodialyzed patients. Free Radic. Biol. Med..

[31] Ceci R., Beltran Valls M.R., Duranti G., Dimauro I., Quaranta F., Pittaluga M., Sabatini S., Caserotti P., Parisi P., Parisi A. (2014). Oxidative stress responses to a graded maximal exercise test in older adults following explosive-type resistance training. Redox Biol..

[32] Ceci R., Duranti G., Leonetti A., Pietropaoli S., Spinozzi F., Marcocci L., Amendola R., Cecconi F., Sabatini S., Mariottini P. (2017). Adaptive responses of heart and skeletal muscle to spermine oxidase overexpression: Evaluation of a new transgenic mouse model. Free Radic. Biol. Med..

[33] Magi F., Dimauro I., Margheritini F., Duranti G., Mercatelli N., Fantini C., Ripani F.R., Sabatini S., Caporossi D. (2018). Telomere length is independently associated with age, oxidative biomarkers, and sport training in skeletal muscle of healthy adult males. Free Radic. Res..

[34] Leonetti A., Baroli G., Fratini E., Pietropaoli S., Marcoli M., Mariottini P., Cervelli M. (2020). Epileptic seizures and oxidative stress in a mouse model over-expressing spermine oxidase. Amino Acids.

[35] Kaminsky L.A., Imboden M.T., Arena R., Myers J. (2017). Reference Standards for Cardiorespiratory Fitness Measured with Cardiopulmonary Exercise Testing Using Cycle Ergometry: Data from the Fitness Registry and the Importance of Exercise National Database (FRIEND) Registry. Mayo Clin. Proc..

[36] Fletcher G.F., Ades P.A., Kligfield P., Arena R., Balady G.J., Bittner V.A., Coke L.A., Fleg J.L., Forman D.E., Gerber T.C. (2013). Exercise standards for testing and training: A scientific statement from the American Heart Association. Circulation.

[37] Powers S.K., Nelson W.B., Hudson M.B. (2011). Exercise-induced oxidative stress in humans: Cause and consequences. Free Radic. Biol. Med..

[38] Bentley D.J., Dank S., Coupland R., Midgley A., Spence I. (2012). Acute antioxidant supplementation improves endurance performance in trained athletes. Res. Sports Med..

[39] Sousa M., Teixeira V.H., Soares J. (2014). Dietary strategies to recover from exercise-induced muscle damage. Int. J. Food Sci. Nutr..

[40] Antonioni A., Fantini C., Dimauro I., Caporossi D. (2019). Redox homeostasis in sport: Do athletes really need antioxidant support?. Res. Sports Med..

[41] Loraschi A., Galli N., Cosentino M. (2014). Dietary supplement and drug use and doping knowledge and attitudes in Italian young elite cyclists. Clin. J. Sport Med..

[42] Mazzarino M., Cesarei L., de la Torre X., Fiacco I., Robach P., Botrè F. (2016). A multi-targeted liquid chromatography-mass spectrometry screening procedure for the detection in human urine of drugs non-prohibited in sport commonly used by the athletes. J. Pharm. Biomed. Anal..

[43] Frajese G.V., Pozzi F., Frajese G. (2006). Tadalafil in the treatment of erectile dysfunction; an overview of the clinical evidence. Clin. Interv. Aging.

[44] Hansen J.M., Choe H.-S., Carney E.W., Harris C. (2001). Differential antioxidant enzyme activities and glutathione content between rat and rabbit conceptuses. Free Radic. Biol. Med..

[45] Thomas T., Thomas T.J. (2001). Polyamines in cell growth and cell death: Molecular mechanisms and therapeutic applications. Cell. Mol. Life Sci..

[46] Salvi M., Toninello A. (2004). Effects of polyamines on mitochondrial Ca(2+) transport. Biochim. Biophys. Acta.

[47] Cervelli M., Amendola R., Polticelli F., Mariottini P. (2012). Spermine oxidase: Ten years after. Amino Acids.

[48] Cervelli M., Angelucci E., Germani F., Amendola R., Mariottini P. (2014). Inflammation, carcinogenesis and neurodegeneration studies in transgenic animal models for polyamine research. Amino Acids.

[49] Rider J.E., Hacker A., Mackintosh C.A., Pegg A.E., Woster P.M., Casero R.A. (2007). Spermine and spermidine mediate protection against oxidative damage caused by hydrogen peroxide. Amino Acids.

[50] Stamler J.S., Singel D.J., Loscalzo J. (1992). Biochemistry of nitric oxide and its redox-activated forms. Science.

[51] Freeman B. (1994). Free radical chemistry of nitric oxide. Looking at the dark side. Chest.

[52] Codoñer-Franch P., Tavárez-Alonso S., Murria-Estal R., Herrera-Martín G., Alonso-Iglesias E. (2011). Polyamines are increased in obese children and are related to markers of oxidative/nitrosative stress and angiogenesis. J. Clin. Endocrinol. Metab..

[53] Di Luigi L., Baldari C., Sgrò P., Emerenziani G.P., Gallotta M.C., Bianchini S., Romanelli F., Pigozzi F., Lenzi A., Guidetti L. (2008). The type 5 phosphodiesterase inhibitor tadalafil influences salivary cortisol, testosterone, and dehydroepiandrosterone sulphate responses to maximal exercise in healthy men. J. Clin. Endocrinol. Metab..

[54] Di Luigi L., Sgrò P., Baldari C., Gallotta M.C., Emerenziani G.P., Crescioli C., Bianchini S., Romanelli F., Lenzi A., Guidetti L. (2012). The phosphodiesterases type 5 inhibitor tadalafil reduces the activation of the hypothalamus-pituitary-adrenal axis in men during cycle ergometric exercise. Am. J. Physiol. Endocrinol. Metab..

[55] Guidetti L., Emerenziani G.P., Gallotta M.C., Pigozzi F., Di Luigi L., Baldari C. (2008). Effect of tadalafil on anaerobic performance indices in healthy athletes. Br. J. Sports Med..

[56] Van Osta A., Moraine J.J., Mélot C., Mairbäurl H., Maggiorini M., Naeije R. (2005). Effects of high altitude exposure on cerebral hemodynamics in normal subjects. Stroke.

[57] Jing Z.C., Yu Z.X., Shen J.Y., Wu B.X., Xu K.F., Zhu X.Y., Pan L., Zhang Z.L., Liu X.Q., Zhang Y.S. (2011). Vardenafil in pulmonary arterial hypertension: A randomized, double-blind, placebo-controlled study. Am. J. Respir. Crit. Care Med..

[58] Leshem E., Caine Y., Rosenberg E., Maaravi Y., Hermesh H., Schwartz E. (2012). Tadalafil and acetazolamide versus acetazolamide for the prevention of severe high-altitude illness. J. Travel. Med..

[59] Zimmermann G.S., von Wulffen W., Huppmann P., Meis T., Ihle F., Geiseler J., Leuchte H.H., Tufman A., Behr J., Neurohr C. (2014). Haemodynamic changes in pulmonary hypertension in patients with interstitial lung disease treated with PDE-5 inhibitors. Respirology.

[60] Sabri M., Beheshtian E. (2014). Comparison of the therapeutic and side effects of tadalafil and sildenafil in children and adolescents with pulmonary arterial hypertension. Pediatr. Cardiol..

[61] Victor R.G., Sweeney H.L., Finkel R., McDonald C.M., Byrne B., Eagle M., Goemans N., Vandenborne K., Dubrovsky A.L., Topaloglu H. (2017). Tadalafil DMD Study Group. A phase 3 randomized placebo-controlled trial of tadalafil for Duchenne muscular dystrophy. Neurology.

[62] McDonald C.M., Sajeev G., Yao Z., McDonnell E., Elfring G., Souza M., Peltz S.W., Darras B.T., Shieh P.B., Cox D.A. (2020). Deflazacort vs prednisone treatment for Duchenne muscular dystrophy: A meta-analysis of disease progression rates in recent multicenter clinical trials. Muscle Nerve.

[63] Sedky A.A., Magdy Y. (2020). Tadalafil versus linaclotide in gastrointestinal dysfunction and depressive behavior in constipation-predominant irritable bowel syndrome. Life Sci..

[64] Pittaluga M., Sgadari A., Dimauro I., Tavazzi B., Parisi P., Caporossi D. (2015). Physical exercise and redox balance in type 2 diabetics: Effects of moderate training on biomarkers of oxidative stress and DNA damage evaluated through comet assay. Oxid. Med. Cell. Longev..

[65] Jackson M.J., Vasilaki A., McArdle A. (2016). Cellular mechanisms underlying oxidative stress in human exercise. Free Radic. Biol. Med..

[66] Ceci R., Duranti G., Di Filippo E.S., Bondi D., Verratti V., Doria C., Caporossi C., Sabatini S., Dimauro I., Pietrangelo T. (2019). Endurance Training Improves Plasma Superoxide Dismutase Activity in Healthy Elderly. Mech. Ageing Dev..

